# Kinetics of Magnesiothermic Reduction of Natural Quartz

**DOI:** 10.3390/ma15196535

**Published:** 2022-09-21

**Authors:** Azam Rasouli, Maria Tsoutsouva, Jafar Safarian, Gabriella Tranell

**Affiliations:** Department of Materials Science and Engineering, Norwegian University of Science and Technology, 7491 Trondheim, Norway

**Keywords:** magnesiothermic reduction, magnesium silicide, silicon, reaction rate, Rietveld refinement

## Abstract

In this work, the kinetics of natural quartz reduction by Mg to produce either Si or Mg_2_Si was studied through quantitative phase analysis. Reduction reaction experiments were performed at various temperatures, reaction times and Mg to SiO_2_ mole ratios of 2 and 4. Rietveld refinement of X-ray diffraction patterns was used to obtain phase distributions in the reacted samples. SEM and EPMA examinations were performed to evaluate the microstructural change during reduction. The results indicated that the reduction reaction rate was slower at a mole ratio of 2 than 4 at the same temperature, as illustrated by the total amount of Si formed (the percent of Si that is reduced to either Si or Mg_2_Si to total amount of Si) being 59% and 75%, respectively, after 240 min reaction time for mole ratios of 2 and 4. At the mole ratio of 4, the reaction rate was strongly dependent on the reaction temperature, where SiO_2_ was completely reduced after 20 min at 1273 K. At the lower temperatures of 1173 and 1073 K, total Si formed was 75% and 39%, respectively, after 240 min reaction time. The results of the current work show that Mg_2_Si can be produced through the magnesiothermic reduction of natural quartz with high yield. The obtained Mg_2_Si can be processed further to produce silane gas as a precursor to high purity Si. The combination of these two processes offers the potential for a more direct and low carbon method to produce Si with high purity.

## 1. Introduction

In order to reduce greenhouse gas emissions, it is of interest to study the feasibility of using alternative reducing agents, such as Al, Mg, CH_4_ and H_2,_ to fossil carbon materials in metal production processes. In this regard, metallothermic reduction of SiO_2_ by Mg as a reducing agent to produce Si with high purity for photovoltaic application offers a CO_2_-free emission process and also potential to introduce less impurity elements into the produced Si [1,2,3,4,5,6,7,8,9,10]. From an economical point of view, using Mg instead of C as the reducing agent currently leads to a more expensive process. However, after reduction, Mg can be recovered from the MgCl_2_ product through carbon-free electrolysis [11,12,13]. In addition, high purity Si is a valuable product compared to metallurgical Si with lower purity, making the use of a more expensive reductant reasonable. Two alternative methods have been proposed to produce pure Si through magnesiothermic reduction: using pure reactants to obtain pure Si or producing Mg_2_Si as an intermediate component to produce SiH_4_ gas that is a precursor to deposit high pure Si [14,15,16,17,18,19,20,21,22,23].

The reaction between Mg and SiO_2_ is fundamentally a simple displacement reaction. Depending on the Mg availability during the reduction reaction, Si or Mg_2_Si are formed, respectively, according to Equations (1) and (2). However, the high reactivity and high vapor pressure of Mg, incomplete reaction or formation of byproducts and high adiabatic temperature of the reduction reaction introduce challenges in terms of feasibility, product yield and large-scale production. Temperature, time, Mg to SiO_2_ mole ratio and silica particle size are variables that determine the reaction rate and consequently product yield [14,15,24,25,26,27,28,29,30,31,32,33,34,35,36,37,38]. It is known from an early study by Wynnyckyi et al. that Mg diffusion is the rate-limiting step during the reduction reaction [31]. However, there is only limited research available in the literature on the topic of reaction rate of the reduction reaction, particularly at temperatures above 1173 K. For example, Gutman et al. reported activation energy around 90 kJ/mol for the reduction of quartz by Mg at temperature range of 723–913 K by using the Jander model [34]. In more recent work, an effective activation energy of 5.5 kJ/mol was obtained at a temperature range of 853–1053 K by applying the Ginstling–Brounshtein model [39]. The former used SiO_2_ disc and the latter used fine powder of SiO_2_ as a Si source.

(1) $$2\mathrm{Mg}+{\mathrm{SiO}}_{2}\to \mathrm{Si}+2\mathrm{MgO}\;\;\Delta H\ll 0$$

(2) $$4\mathrm{Mg}+{\mathrm{SiO}}_{2}\to {\mathrm{Mg}}_{2}\mathrm{Si}+2\mathrm{MgO}\;\;\Delta H\ll 0$$

In our previous work [40], it was demonstrated how the reaction conditions including Mg to SiO_2_ mole ratio, SiO_2_ particle size and quartz type affect the reduction of natural quartz. Complementary to our previous work, the aim of the current work was to investigate the reaction rate of the magnesiothermic reduction of natural quartz to produce either Si or Mg_2_Si at various temperatures. The reaction rate studies were supported by X-ray diffraction-based quantitative phase analysis (QPA) using the Rietveld method.

## 2. Materials and Methods

Natural quartz, with a composition given in Table 1, was used as the silica source. For the reducing agent, Mg turnings (≥99.8 wt%, size range of ≤3.2 mm, Alfa Aesar, Kandel, Germany) were chosen. In each experiment, around 0.6 g of SiO_2_ with a particle size of 3360 ± 780 µm and the stoichiometric amount of Mg turnings to conduct reactions in Equations (1) and (2) were used. Three different temperatures (1073, 1173 and 1273 K) were chosen for conducting experiments. At each reaction temperature, a series of experiments with varying reaction times from 10 to 240 min was performed. The experimental procedure to conduct the magnesiothermic reduction was described in detail in our previous publication [40].

To identify phases in the reacted samples, X-ray diffraction analyses were performed using a Bruker D8 Focus (Bruker AXS GmbH, Karlsruhe, Germany) with CuKα radiation (wavelength of 1.54 Å) and LynxEye™ SuperSpeed Detector. The samples were back loaded into the sample holders and horizontally spun during measurement to improve particle statistics. Diffractometer scans were recorded in Bragg–Brentano geometry from 5 to 115° 2θ, with a step size of 0.016° 2θ and 2 s counting time per step. After obtaining X-ray diffraction patterns, the phases were identified by DIFFRAC.EVA software (V5.2, Bruker AXS GmbH, Karlsruhe, Germany) by using the PDF-4+ database (2014, ICDD, Philadelphia, Pennsylvania, USA) [41,42]. The powder diffraction files (PDF) of detected phases are 00-046-1045 for SiO_2_, 00-035-0821 for Mg, 04-010-4039 for MgO, 04-001-7247 for Si, 00-035-0773 for Mg_2_Si and 01-083-5238 for Mg_1.98_Si. The progress of reaction at various reaction times was studied through quantitative phase analysis using Topas software (V5, Bruker AXS GmbH, Karlsruhe, Germany) based on Rietveld method [43,44]. Before running the refinement in Topas software, some parameters were refined to accomplish better fitting. In the background item, the “Chebyshev polynomial” of 7th order was selected. In the corrections item, the “Zero error” and “LP factor” were fixed at 0, while “sample displacement” was chosen to be refined. Finally, for the Mg_2_Si and Mg_1.98_Si phases, “Preferred Orientation” for “Direction 1” was refined. The refinement result for one sample is given in the supplementary material Figure S1, that illustrates a good fit. For all analyzed samples, weighted profile R-factor, R_WP,_ was smaller than 10 and goodness of fit, GOF, was in the range of 3.2 to 3.8. To evaluate the accuracy of the method, quantitative phase analysis was also performed on samples with known chemical composition. To prepare the standard samples, different mixtures of SiO_2_ (natural quartz with composition given in Table 1), Mg (≥99.8 wt%, Alfa Aesar, Kandel, Germany), Si, Mg_2_Si (≥99.5%, Thermo Scientific, Kandel, Germany) and MgO (assay ≥ 97%, Merk, Darmstadt, Germany) were made. The material mixes were milled by using a WC vibratory disk mill (RS 200, RETSCH GmbH, Haan, Germany) for 30 s at 900 rpm speed to obtain homogeneous samples. It is worth mentioning that to achieve finer particles, it was found to be beneficial to add a few drops of acetone to the powder mixture before milling. The known chemical compositions and the ones obtained by quantitative X-ray phase analysis are compared in Table S1. The comparison illustrates a good agreement between the known chemical composition and the calculated ones with a maximum observed difference between the known chemical compositions and the calculated ones of 3.4 wt%. For simplicity, the sum of Mg_2_Si and Mg_1.98_Si phases are reported as produced Mg_2_Si during the reduction reaction. However, during refinement in Topas software, both of these phases were considered to obtain better fit.

**Table 1:** Chemical composition of natural quartz analyzed by inductively coupled plasma–optical emission spectrometry (ICP-OES) (the analysis was performed by Jusnes [45]).

Elements	Al	Fe	K	Mg	Na	Ca	Ti	Mn	P	SiO_2_
amount (ppm)	106	20	23	9	10	3	4.2	0.3	<2.6	balance

Microstructural examinations of the reacted samples were performed by Field Emission Scanning Electron Microscopy (Zeiss Ultra FESEM, Carl Zeiss AG, Oberkochen, Germany). In addition, chemical composition analysis and X-ray mapping were carried out using Electron Probe Micro analyzer (EPMA) (JXA-8500F, JEOL Ltd., Akishima, Japan).

## 3. Results

### 3.1. Distribution of Phases

**Figure 1:**
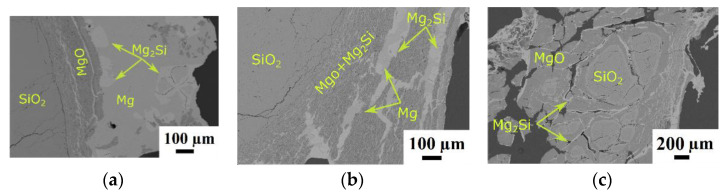
Backscattered electron images of samples at a Mg/SiO_2_ mole ratio of 2, 1173 K and different reaction times: (**a**) 10 min; (**b**) 20 min; (**c**) and 120 min.

The microstructure of partially reduced SiO_2_ after reduction for various reaction times at a Mg/SiO_2_ mole ratio of 2 and 1173 K is shown in Figure 1. After 10 min reaction time, Figure 1a, a mixture of MgO and Mg_2_Si as product phases, are found around the quartz particle. In addition, unreacted Mg is observed adjacent to MgO and Mg_2_Si products. By increasing the reaction time to 20 min, the thickness of the product layer around the unreacted SiO_2_ core increased. In the product layer, Mg is found in association with Mg_2_Si as thin bands (in a 2D image) in the MgO matrix, as seen in Figure 1b. There are already some inherent cracks in the quartz particles, but more cracks are formed during the reduction reaction. As illustrated in Figure 1c, quartz particles are fractured into smaller pieces after 120 min reaction time. Diffusion of Mg through particles and conversion of SiO_2_ to MgO and Mg_2_Si product phases is accompanied by a volume expansion of approximately 138% at 1173 K, calculated by FactSage software (8,1, Thermfact/CRCT, Montreal, Quebec, Canada & TT-Technologies, Aachen, Germany) [46]. This large volume expansion introduces stress inside the particles and, in addition to the significant heat released during the exothermic reaction as discussed later in Section 4.2, are factors that facilitate the cracking.

**Figure 2:**
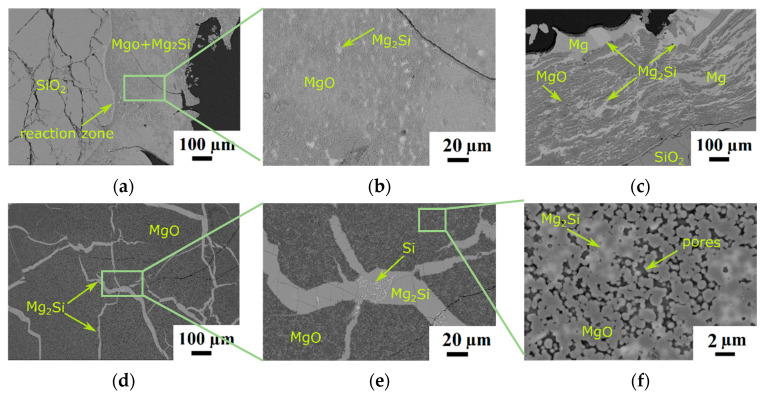
Backscattered electron images of samples at a Mg/SiO_2_ mole ratio of 4 and different temperatures: (**a**) 1073 K and 120 min; (**b**) higher magnification of the area shown in the rectangle in Figure 2a; (**c**) 1173 K and 20 min; (**d**) 1273 K and 20 min; (**e**) higher magnification of the area shown in the rectangle in Figure 2d; (**f**) higher magnification of the area shown in the rectangle in Figure 2e.

Reduced SiO_2_ particles at a Mg/SiO_2_ mole ratio of 4 display a very different microstructure at different reaction temperatures, as seen in Figure 2. At temperature of 1073 K, Figure 2a,b, the Mg_2_Si product was formed as small particles in the MgO matrix. At 1173 K, the Mg_2_Si and Mg phases are observed as thin bands in the MgO matrix (in a 2D image), Figure 2c. This microstructure is similar to the microstructure observed at the mole ratio of 2 and same temperature in Figure 1b but with a higher amount of metal phase. At the higher temperature of 1273 K, the Mg_2_Si product phase separated from the MgO phase either to the external surface of the particle or between the fractured MgO particles that were originally a SiO_2_ phase, Figure 2d. By Mg_2_Si separation, the MgO phase developed a porous structure, as seen in Figure 2e,f.

**Figure 3:**
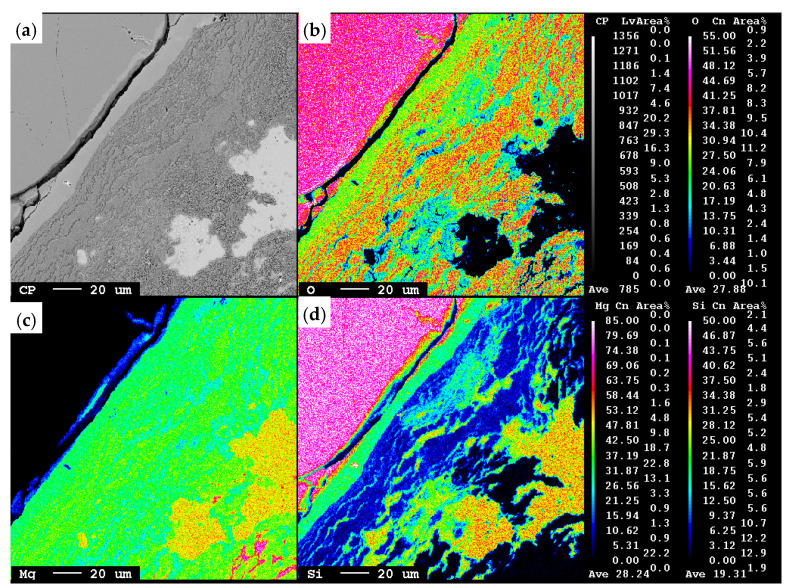
(**a**) Backscattered electron image of the interface between SiO_2_ and products of the sample at a Mg/SiO_2_ mole ratio of 4, 173 K temperature and 20 min reaction time; (**b**) X-ray mapping of O; (**c**) X-ray mapping of Mg; (**d**) X-ray mapping of Si.

Figure 3 is a high magnification image of the microstructure of the reaction zone between the SiO_2_ particle and the product layer at a Mg/SiO_2_ mole ratio of 4 and 1173 K. As can be seen in the X-ray mapping of different elements, various layers with different chemical compositions were formed around the quartz particle. Point analysis reveals that the thin gray layer around the SiO_2_ core is composed of two layers. The innermost layer has a chemical composition close to Mg_2_SiO_4_, and the second layer contains MgO and Si. The next layer, of dark gray color, is a mixture of MgO and Mg_2_Si. In the outermost layer, MgO and Mg_2_Si appear as separate phases. Moreover, some pure Mg is found in this layer, shown as pink areas in the X-ray mapping of Mg, Figure 3c. The formation of these layers is in accordance with the ternary phase diagram of Si–Mg–O that dictates the establishment of possible interfaces between different phases. These observations imply that the conversion of SiO_2_ to Mg_2_Si happens through a series of intermediate reactions as discussed in detail in our previous work [40].

### 3.2. Rate of Reduction Reaction

**Figure 4:**
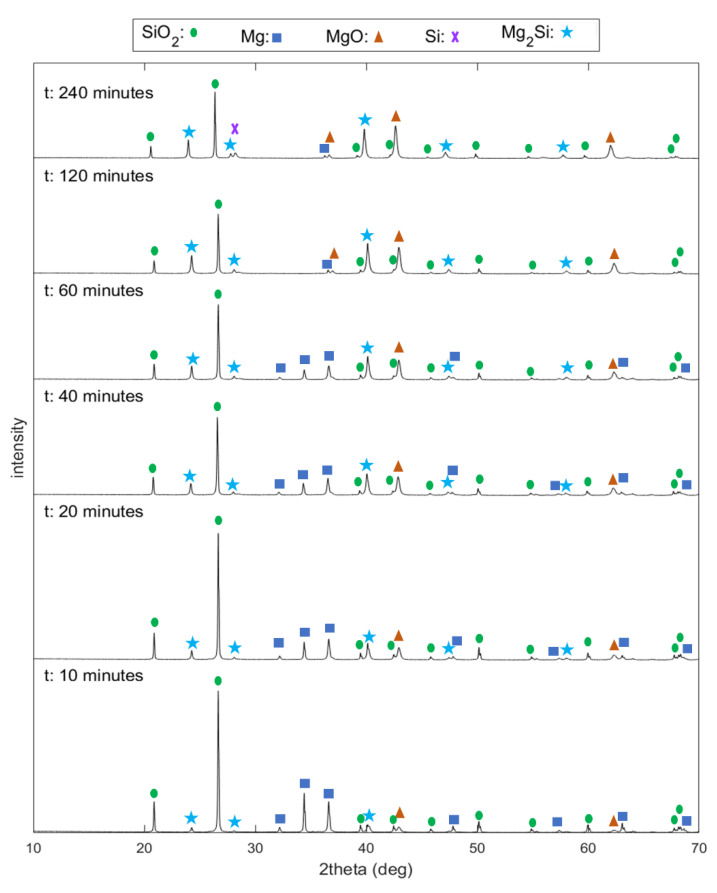
X-ray diffraction patterns at a Mg/SiO_2_ mole ratio of 2, 1173 K and various reaction times.

X-ray diffraction patterns of samples at a Mg/SiO_2_ mole ratio of 2 and different reaction times are given in Figure 4. X-ray diffraction patterns of other samples can be found in Figures S2–S4. It is observed that small peaks of product phases appear after 10 min reaction time. With increasing the reaction time, the intensities of these peaks increase while the peak intensities of reactants decrease. 

#### 3.2.1. Effect of Mg/SiO_2_ Mole Ratio

**Figure 5:**
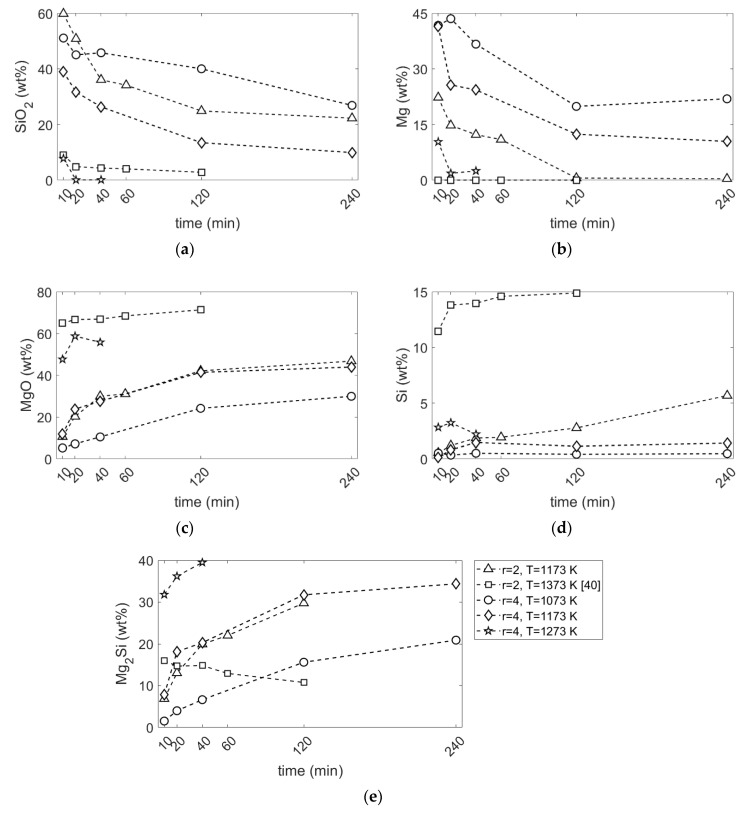
Changes in the amount of different phases against reaction time: (**a**) SiO_2_; (**b**) Mg; (**c**) MgO; (**d**) Si; (**e**) Mg_2_Si; (r is the Mg/SiO_2_ mole ratio).

It is clearly seen that the amounts of SiO_2_ and Mg phases decrease, Figure 5a,b, while MgO, Si and Mg_2_Si amounts increase, Figure 5c,d, as a function of reaction time. Generally, the rate of change in the amounts of phases are faster at the beginning, implying a higher initial reaction rate. This behavior is observed for diffusion-controlled reactions where the increasing thickness of the product layer results in a slower reaction rate with time [47].

At a Mg/SiO_2_ mole ratio of 2, where Mg and SiO_2_ react according to Equation (1) to produce MgO and Si, only a small amount of Si was formed: 6 wt% at 1173 K after 240 min, as seen in Figure 5d. The equilibrium amount of Si for complete reduction is 24 wt%. Instead, a significant amount of Mg_2_Si was formed, as shown in Figure 5e. The Mg_2_Si content is increasing until it reaches approximately to 30 wt% at 120 min reaction time. After this time, the amount of Mg_2_Si starts to decline. As seen in Figure 5a,b, there is still approximately 25 wt% SiO_2_ left unreacted, while there is no Mg left after 120 min reaction time. Hence, after all Mg was converted to MgO or combined with Si to form Mg_2_Si, the reduction reaction progresses by supplying Mg from Mg_2_Si phase. The rate of Si formation becomes slightly faster after this point since Si was formed through two reactions: SiO_2_ reduction and conversion of Mg_2_Si to Si.

**Figure 6:**
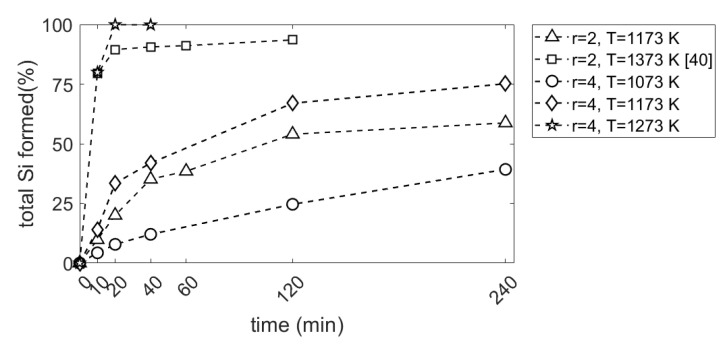
Total Si formed against reaction time (r is the Mg/SiO_2_ mole ratio).

At a Mg/SiO_2_ mole ratio of 4 and 1173 K temperature, X-ray diffraction patterns in Figure S3 show Mg_2_Si as the main product, which is in accordance with products in Equation (2). Rietveld phase analysis results in Figure 5e also show that the amount of Mg_2_Si is always increasing with reaction time and reaches approximately 34 wt% after 240 min reaction time. Only a small amount of Si was formed; the Si content increases up to approximately 1.5 wt% after 40 min reaction time, where it remains approximately constant until 240 min reaction time, as shown in Figure 5d. To compare the total reduced SiO_2_ to either Si or Mg_2_Si, total Si formed is defined according to Equation (3):

(3) $$\mathrm{total}\;\mathrm{Si}\;\mathrm{formed}\;(\%)=\frac{{\mathrm{Si}}_{\mathrm{si}}+{\mathrm{Si}}_{{\mathrm{Mg}}_{2}\mathrm{Si}}}{{\mathrm{Si}}_{\mathrm{si}}+{\mathrm{Si}}_{{\mathrm{Mg}}_{2}\mathrm{Si}}+{\mathrm{Si}}_{{\mathrm{SiO}}_{2}}}\times 100$$

where Si_Si_, ${\mathrm{Si}}_{{\mathrm{Mg}}_{2}\mathrm{Si}}$ and ${\mathrm{Si}}_{{\mathrm{SiO}}_{2}}$ are the amounts of Si in Si, Mg_2_Si and SiO_2_ phases, respectively, in wt%. It is seen in Figure 6 that the total Si formed is slightly higher for the Mg/SiO_2_ mole ratio of 4 than for 2 until 40 min reaction time at a temperature of 1173 K. After this point, the difference between the total Si formed at Mg/SiO_2_ mole ratios of 2 and 4 becomes more significant. After 240 min reaction time, the total Si formed is 59 and 75 wt%, respectively, for Mg/SiO_2_ mole ratios of 2 and 4. This shows that the amount of available Mg affects the reaction rate as is further discussed in Section 4.1. The comparison of total Si formed at a Mg/SiO_2_ mole ratio of 2 and 1173 K with results at 1373 K from our previous work [40] reveals that reaction rate is considerably higher at 1373 K than 1173 K.

#### 3.2.2. Effect of Temperature

The comparison of residual SiO_2_ at 1073, 1173 and 1273 K as a function of time in Figure 5a reveals that the reaction rate is strongly dependent on the reaction temperature at a Mg/SiO_2_ mole ratio of 4. While all SiO_2_ was reduced to either Si or Mg_2_Si phases after 20 min reaction time at 1273 K, there is still a noticeable amount of SiO_2_ left at 1073 and 1173 K—45 and 32 wt%, respectively.

The amount of produced Si at a Mg/SiO_2_ mole ratio of 4 where SiO_2_ and Mg reacts according to Equation (2) to form Mg_2_Si and MgO increases slightly with increasing the reaction temperature. There is approximately 0.5, 1.5 and 3.2 wt% Si at 1073, 1173 and 1273 K, respectively, as seen in Figure 5d. With more SiO_2_ reduction, more Si was formed at this Mg/SiO_2_ mole ratio.

## 4. Discussion

### 4.1. Reaction Rate

Reduction of SiO_2_ particles by Mg can be described by the shrinking untreated core model where the reduction reaction starts at the surface of SiO_2_ particles and progresses by diffusion of Mg through the formed product layer(s) to reach the unreacted SiO_2_ core. This reaction consists of three steps: (1) mass transport of Mg from the bulk of Mg to the external surface of quartz particles, (2) diffusion of Mg through the product layer(s) towards the reaction zone and (3) chemical reaction at the reaction zones between silicate layers/SiO_2_ and Mg. It was shown earlier that diffusion of Mg through the product layer toward the reaction zone is the rate limiting step [31,34,35,37,40]. 

The vapor pressure of Mg and the number of moles of Mg in the gas phase at different reaction temperatures, are calculated by Equations (4) and (5), respectively [48].

(4) $${\mathrm{Mg}}_{\left(l\right)}{=\mathrm{Mg}}_{\left(g\right)}\;\;∆G\text{°}=-\mathrm{RTln}\frac{P_{\mathrm{Mg}}}{a_{\mathrm{Mg}}}$$

**Figure 7:**
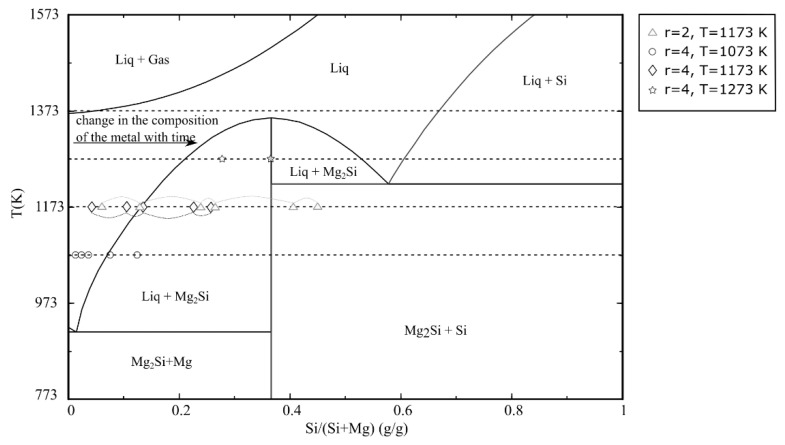
Binary phase diagram of Mg–Si, plotted by FactSage 8.1(r is the Mg/SiO_2_ mole ratio).

(5) PV=nRT

where ΔG° is the standard Gibbs free energy, R is the universal gas constant, T is the temperature, P_Mg_ is the Mg vapor pressure and a_Mg_ is the activity of Mg that is equal to 1, P is the gas pressure, V is the volume of the system and n is the number of moles of gas phase. In Equation (5), it is assumed that the Mg vapor is an ideal gas. Calculated values in Table 2 reveal that despite a relatively high vapor pressure of Mg, only a small fraction of the total Mg is present in the gas phase. This is due to the small volume of the closed reactor used in this study. It can hence be assumed that there are three main phases present during reaction, SiO_2_ and MgO oxides as two solid phases and a metal phase that contains Mg and Si elements. The composition of the metal phase is changing over the reaction time, as shown in the binary phase diagram of Si–Mg in Figure 7. At a reaction time of zero, the metal phase is composed of pure Mg. By Mg consumption during the reaction, the metal composition changes along the isothermal lines. In other words, it changes from a liquid region to a mixed liquid and solid (Mg_2_Si) region with a final composition of Mg_2_Si for complete reaction at a Mg/SiO_2_ mole ratio of 4. At a mole ratio of 2, the change in metal composition progresses to the solid region with a Mg_2_Si and Si mixture until to the metal phase is entirely composed of Si. Therefore, the metal phase is completely solid after it contains more than 36.6 wt% Si. This can lead to lower reaction rate at mole ratio of 2 when there is no liquid metal. It is worth mentioning that it is assumed that the metal has a uniform composition throughout the particle. 

The Ginstling–Brounshtein model and the first-order reaction model are described, respectively, by Equations (6) and (7) [49,50].

(6) $$1-\left(\frac{2}{3}\right)\alpha -{\left(1-\alpha \right)}^{\frac{2}{3}}=kt$$

(7) $$-ln\left(1-\alpha \right)=kt$$

where *α* is the conversion fraction and *k* is the rate constant. The conversion fraction and the rate constant can be expressed by Equations (8) and (9), respectively:

(8) $$\alpha =\frac{m_{0}-m_{t}}{m_{0}-m_{\infty }}$$

**Figure 8:**
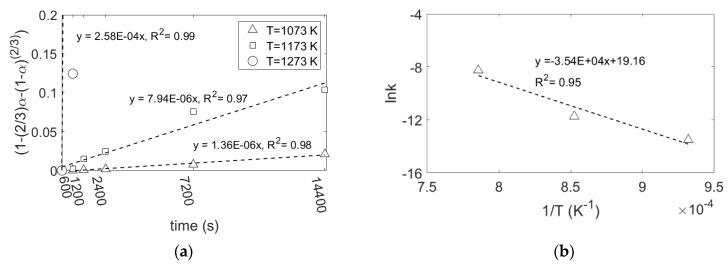
(**a**) Right-hand side of Equation (6) against reaction time at different reaction temperatures, Ginstling–Brounshtein model; (**b**) lnk against 1/T by using Equation (9).

**Figure 9:**
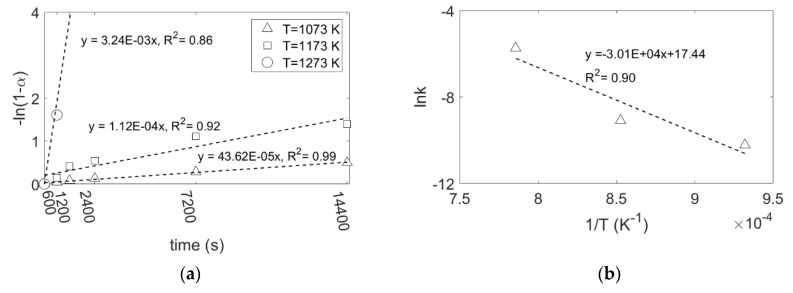
(**a**) Right-hand side of Equation (7) against reaction time at different reaction temperatures, first-order reaction model; (**b**) lnk against 1/T by using Equation (9).

(9) k=k0exp−EaRT

where *m*_0_ is the initial weight of SiO_2_, *m_t_* is the weight of SiO_2_ at time *t*, *m_∞_* is the final weight of SiO_2_ that here is equal to zero, *k*_0_ is the frequency factor, *E_a_* is the activation energy, *R* and *T* have been defined earlier. By plotting the right-hand side of Equations (6) and (7) against reaction time, the rate constant at different temperatures is obtained, as shown in Figure 8a and Figure 9a, respectively. Furthermore, by applying Equation (9), the activation energy of the reduction reaction at a Mg/SiO_2_ mole ratio of 4, is calculated as 294 and 250 kJ/mol, respectively, for the graphs shown in Figure 8b and Figure 9b. The activation energy obtained from the two reaction rate models are in a good agreement. These values are comparable with the activation energy of Mg diffusion in MgO, 266.3 kJ/mol and 215 kJ/mol, reported respectively by Wuensch et al. and Martinelli et al. [51,52], further supporting Mg diffusion in MgO as the rate limiting step. As mentioned earlier, MgO exhibits different microstructures depending on the reaction temperature, particularly at 1273 K, which is expected to affect the diffusion rate of Mg. However, it would not change the activation energy of the reaction to a value lower than the one for Mg diffusion in MgO.

### 4.2. Evaluation of “True” Internal Sample Temperature during Reduction Reaction

As the magnesiothermic reduction of SiO_2_ is strongly exothermic, it may be reasonable to assume that the actual temperature generated inside the reactor as well as inside the particles, is considerably higher than the set-point temperature. To measure the “macro” temperature change during the reduction reaction, an experiment was performed where a monitoring thermocouple was set directly in contact with quartz particles by using an open reactor. This set-up and the set-up for the closed reactor are shown in Figure S5a,b. The results in Figure S5c show that no significant temperature change, i.e., on the sample level, was observed as the measured temperature was very close to the set reaction temperature of 1073 K. The adiabatic temperature is also calculated by HSC Chemistry software (9, Metso Qutotec, Tampere, Finland) [53] in Table 3 and reveals that the reaction in Equation (2) results in high adiabatic temperatures at the three reaction temperatures for both complete reduction of SiO_2_ and partial SiO_2_ reduction, corresponding to reduction at 1073 and 1173 K for 240 min reaction time. While according to calculations the temperature of the system including sample, Al_2_O_3_ crucible and stainless-steel reactor should only increases slightly after reduction of SiO_2_ at 1073, 1173 and 1273 K. This indicates that the amount of released heat was too small to rise the temperature of the entire system noticeably.

However, released heat at the reaction interface could potentially lead to localized high temperatures. To theoretically evaluate the temperature profile inside the sample, a spherical quartz particle with the weight equals to the average weight of particles used in the experiments is assumed to be reduced at the reaction rate equals to the reduction of SiO_2_ in Figure 5a at a Mg/SiO_2_ mole ratio of 4. To be able to derive equations to obtain the temperature profile, the following assumptions were made:

- It is an adiabatic system that contains one quartz particle. Therefore, heat radiation and heat convection from the surface of the particle are assumed to be zero.
- Reactions progress uniformly from the surface of the particle towards the core. Therefore, the three-dimensional problem is converted to a one-dimensional problem in regard to symmetry.
- The released heat during reduction increases the temperature at the reaction zone to the adiabatic temperature of reaction.
- The heat capacity of each phase was obtained from HSC Chemistry 9 software. Two other temperature dependent properties, heat conductivity and density were calculated by FactSage 8.1 software. At higher temperatures where these properties are not available, extrapolation from the available data was applied. The heat capacity of phases is given in Table S2 and heat conductivity and density are listed in Table S3.
- There is an ideal interface between the reactant and products that the heat conducts along.
- The thicknesses of the reaction zone were obtained from the microstructure examination, as shown in Figure 3.

**Figure 10:**
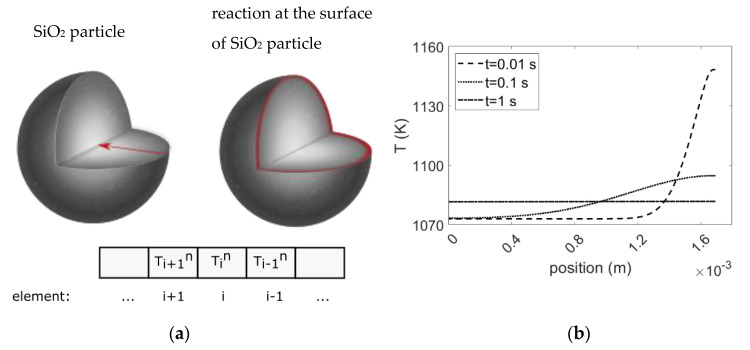
(**a**) Schematic illustration of heat transfer through a particle radius. (**b**) Temperature profile after reduction of first element at a Mg/SiO_2_ mole ratio of 4 and 1073 K.

The law of conversion of energy is described by Equation (10) when only conduction heat transfer take places [54]. For a small element (*i*) with Δ*x* thickness in Figure 10a, Equation (10) can be written according to Equation (11):

(10) $$\frac{\partial }{\partial x^{2}}\left(-k_{m}\frac{\partial T}{\partial x}\right)=\rho_{m}c_{\rho m}\frac{\partial T}{\partial t}$$

(11) $${\left(-k_{m}\frac{T_{i}^{n}-T_{i+1}^{n}}{∆x}\right)}_{x}-{\left(-k_{m}\frac{T_{i-1}^{n}-T_{i}^{n}}{∆x}\right)}_{x+∆x}=\rho_{m}c_{\rho m}∆x\frac{T_{i}^{n+1}-T_{i}^{n}}{∆t}$$

where *x* is the position, Δ*x* is the element thickness that equals to the reaction zone thickness, *t* is the time, $T_{i}^{n}$ is the temperature of element *i* at *t = n*, $T_{i+1}^{n}$ is the temperature of element *I + 1* at *t = n,* $T_{i}^{n+1}$ is the temperature of element *i* at *t = n + 1, k_m_* is the thermal conductivity of phase *m*, *ρ_m_* is the density of phase *m* and *c_p,m_* is the heat capacity of phase *m*.

Equation (11) was solved for one dimensional problem with length equals to the radius of a particle using MATLAB R2021a software (The MathWorks, Inc., Natick, MA, USA) [55]. At *t* = 0 s, all elements are composed of SiO_2_ at the reaction temperature. It is assumed that at *t =* 10^−5^ s (equals to the time step) the first element at the particle surface is converted to the product phases with temperature equals to the adiabatic temperature. Under real conditions, the reaction zone moves gradually; for example it takes 147 s for the reaction zone to move from the surface of the particle to the interface between the first and second elements at a reaction temperature of 1073 K. After reduction of the first element, the second element reacts with Mg and its temperature increases to the adiabatic temperature of reaction, while the temperatures of other elements increase slightly because of the released heat during reduction of the first element. In this way, the reaction zone continues to move towards the core of the particle by progression of the reaction. As can be seen in Figure 10b the released heat is rapidly transferred after reduction of the first element. It takes approximately 1 s to obtain a uniform temperature throughout the particle. Moreover, the product side has a higher temperature than the SiO_2_ side given the higher thermal conductivity of MgO compared to SiO_2_.

It can hence be concluded that there would be a local, high temperature in the reaction zone or in the vicinity of it. However, the released heat is dissipated rapidly throughout the particle. Consequently, the overall system temperature would remain close to the set reaction temperature.

## 5. Conclusions

In this work, the reduction reaction rate of natural SiO_2_ particles by Mg to produce either Si or Mg_2_Si was studied as a function of temperature (1073–1273 K) and Mg/SiO_2_ mole ratio (2 and 4). The main conclusions of the present work are summarized as:

- The activation energy of the reduction reaction at a Mg/SiO_2_ mole ratio 4 was determined to be 294 and 250 kJ/mol, using the Ginstling–Brounshtein model and the first order reaction model, respectively. The calculated activation energy confirms that the Mg diffusion through the MgO-based product layer controls the reaction rate.
- A lower reaction rate at a Mg/SiO_2_ mole ratio of 2 than 4 can be attributed to the state of the metal phase that is in solid region for the latter mole ratio.
- The microstructure examination shows that the product layer has very different microstructures under different reaction conditions with respect to the distribution of product phases (MgO and metal).
- Despite the strongly exothermic reaction giving a high localized temperature at the reaction interface, the bulk temperature of the particle system did not increase substantially given the size of the current system and the high rate of heat transfer.

## Figures and Tables

**Table 2:** Vapor pressure and number of Mg moles in the gas phase; (vapor pressure is calculated by FactSage 8.1).

Parameters	T (K)			
	1273	1173	1273	1373
Vapor pressure of Mg (Pa)	4.39 × 10^3^	1.54 × 10^4^	4.37 × 10^4^	1.06 × 10^5^
Number of Mg moles in the gas phase	5.26 × 10^−6^	1.68 × 10^−5^	4.42 × 10^−5^	9.91 × 10^−5^
Percent of Mg moles in the gas phase	0.16	0.50	1.31	2.93

**Table 3:** Temperature increment at different reaction conditions, calculated by HSC 9 software (temperatures are given in K unit).

Set Reaction Temperature	T_ad_ for Complete Reaction	T_ad_ after 4 h Reaction Time	Temperature of the System Including Reactant Material, Al_2_O_3_ Crucible and Stainless-Steel Reactor
1073	2663	1694	1074
1173	2759	2366	1174
1273	2854	2854	1275

## Data Availability

Not applicable.

## Supplementary Materials

The following supporting information can be downloaded at: https://www.mdpi.com/article/10.3390/ma15196535/s1, Table S1: The comparison of chemical compositions of samples with known compositions and values obtained from quantitative phase analysis; Table S2: Heat capacity of phases obtained from HSC Chemistry 9 software; Table S3: Density and thermal conductivity of phases obtained from FactSage 8.1 software; Figure S1: Rietveld refinement result of the sample with a Mg/SiO2 mole ratio of 4, 1173 K and 240 min reaction time; Figure S2: X-ray diffraction patterns at a Mg/SiO2 mole ratio of 4, 1073 K and various reaction times; Figure S3: X-ray diffraction patterns at a Mg/SiO2 mole ratio of 4, 1173 K and various reaction times; Figure S4: X-ray diffraction patterns at a Mg/SiO2 mole ratio of 4, 1273 K and various reaction times; Figure S5: (a) Closed reactor used to conduct experiments, (b) open reactor to measure temperature close to reactants during a reduction reaction, (c) measured temperature against reaction time for two set-ups.
