# Supplementary material for: Kinetics of Magnesiothermic Reduction of Natural Quartz
Source: Materials (Basel). 2022 Sep 21;15(19):6535. doi: 10.3390/ma15196535 (PMC9571835; doi:10.3390/ma15196535)
Supplement: Supplementary file 1 [file materials-15-06535-s001.zip › materials-1897882-supplementary.pdf]

Supplementary material for:

Kinetics of Magnesiothermic Reduction of Natural Quartz

Azam Rasouli *, Maria Tsoutsouva, Jafar Safarian and Gabriella Tranell

Department of Materials Science and Engineering, Norwegian University of Science and Technology, 7491 Trondheim, Norway
* Correspondence: azam.rasouli@ntnu.no

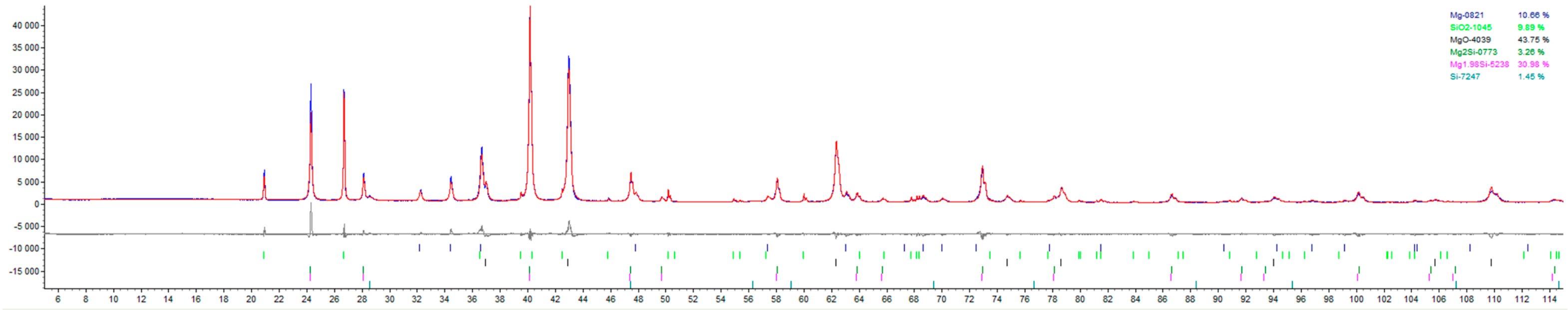

Figure S1. Rietveld refinement result of the sample with a Mg/SiO_2 mole ratio of 4, 1173 K and 240 minutes reaction time.

Table S1. The comparison of chemical compositions of samples with known compositions and values obtained from quantitative phase analysis.

phases	Chemical composition (wt%)								
	Sample 1			Sample 2			Sample 3		
	Known	QPA	difference	Known	QPA	difference	Known	QPA	difference
SiO_2	34.3	37.7	−3.4	38.9	41.9	−3.0	23.5	26.9	−3.4
Mg	-	-	-	61.1	58.1	3.0	2.2	2.0	0.2
Si	10.3	8.3	2.0	-	-	-	23.7	22.3	1.4
Mg_2Si	-	-	-	-	-	-	2.2	1.8	0.4
MgO	55.4	54.0	1.4	-	-	-	48.3	47.1	1.2

phases	Chemical composition (wt%)								
	Sample 4			Sample 5			Sample 6		
	Known	QPA	difference	Known	QPA	difference	Known	QPA	difference
SiO_2	11.2	13.5	−2.3	-	-	-	-	-	-
Mg	10.3	9.1	1.2	2.0	2.5	−0.5	-	-	-
Si	2.2	2.4	−0.2	-	-	-	25.7	24.7	1.0
Mg_2Si	30.1	29.5	0.6	47.4	44.5	2.9	-	-	-
MgO	46.1	45.5	0.6	50.6	53.1	−2.5	74.3	75.3	−1

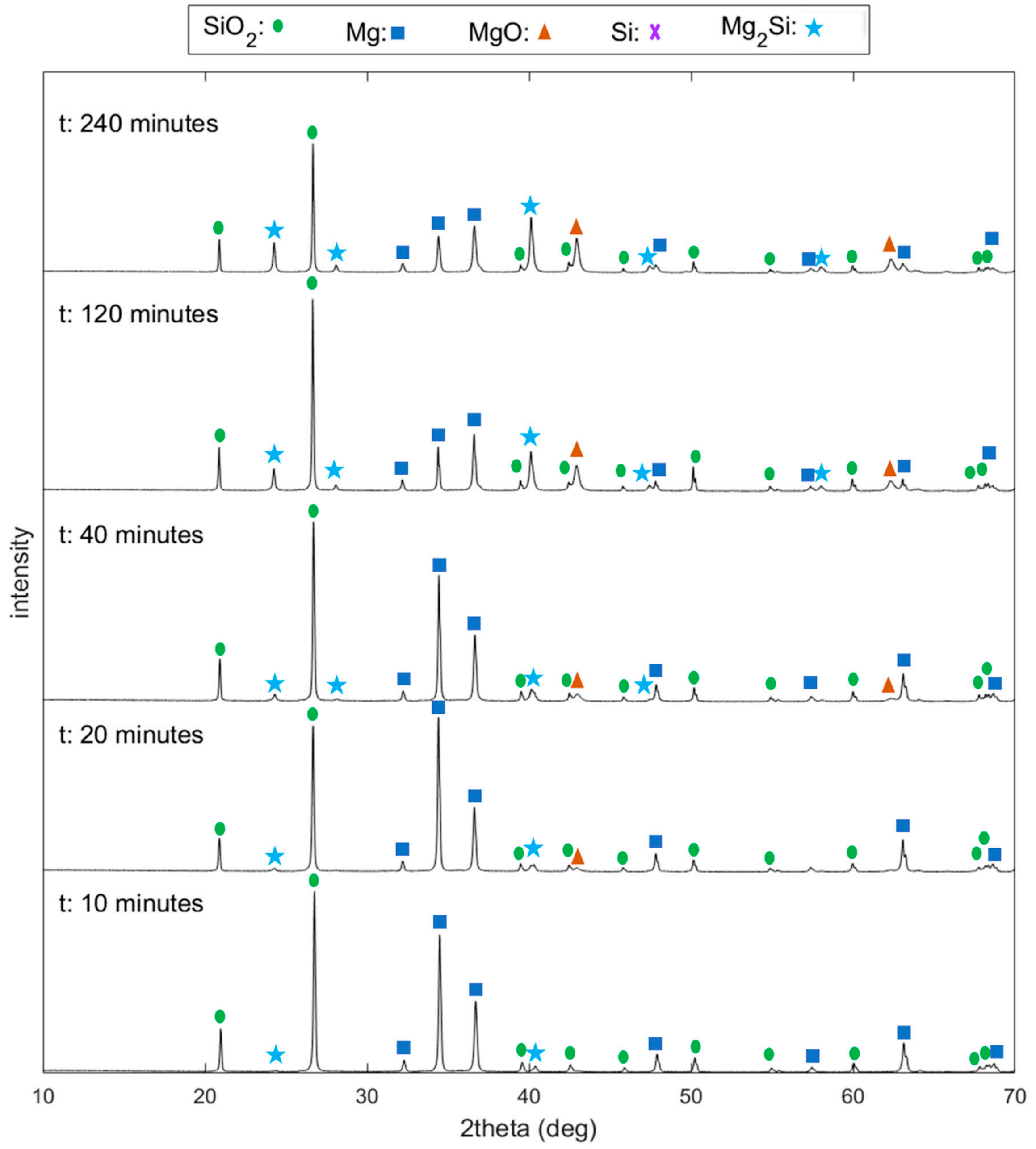

Figure S2. X-ray diffraction patterns at a Mg/SiO_2 mole ratio of 4, 1073 K and various reaction times.

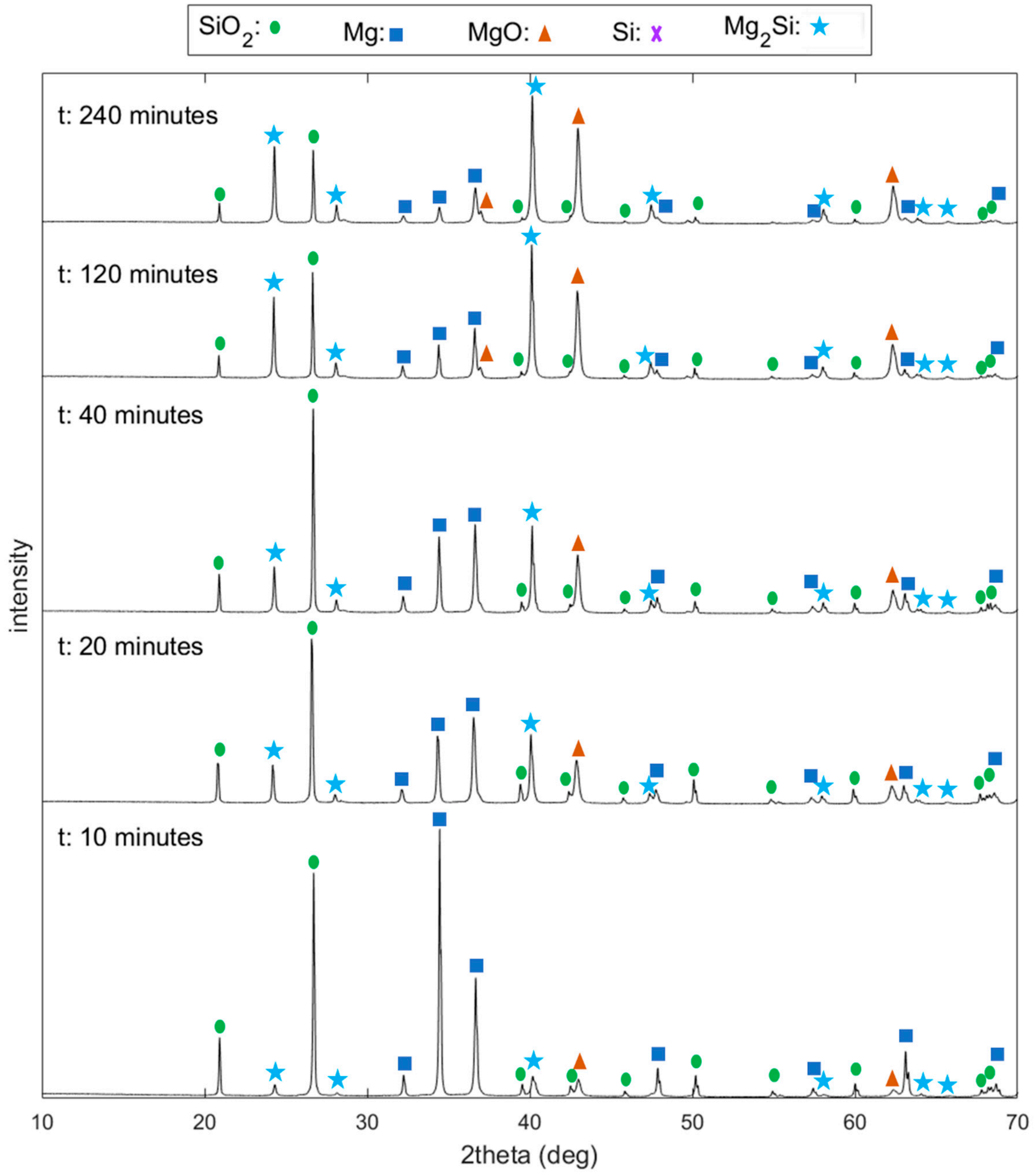

Figure S3. X-ray diffraction patterns at a Mg/SiO_2 mole ratio of 4, 1173 K and various reaction times.

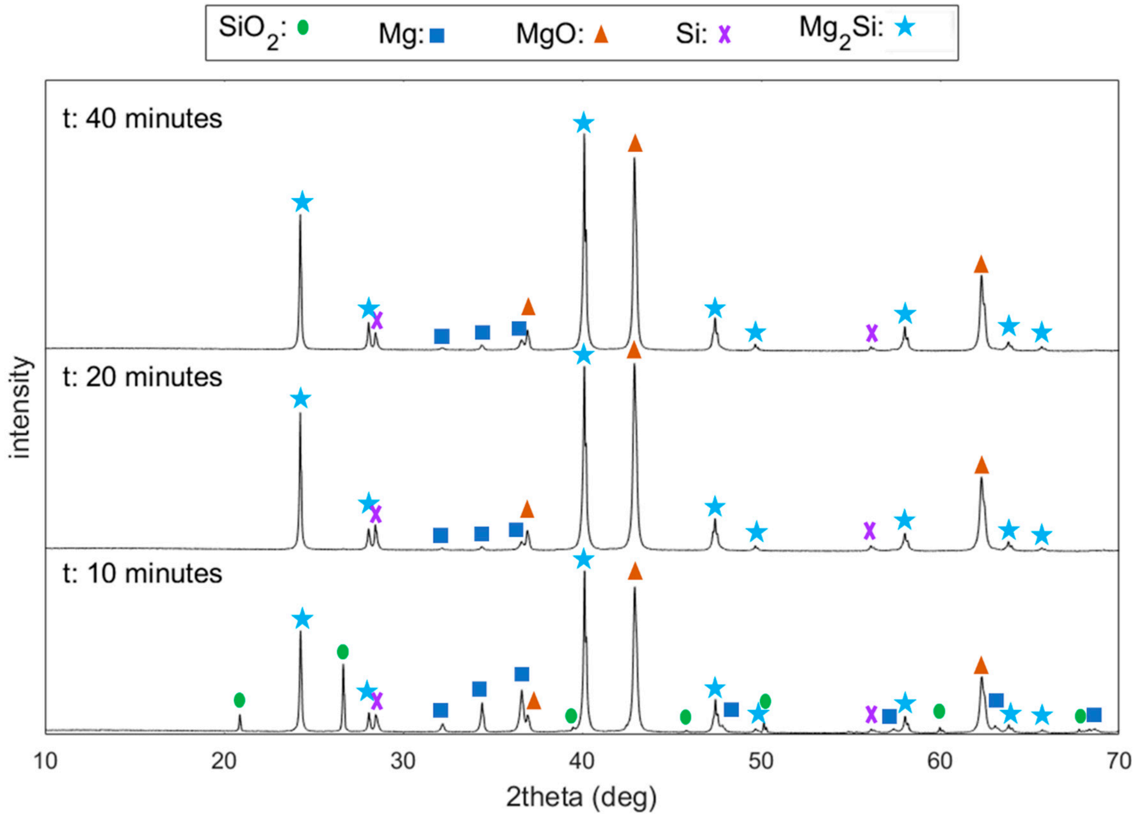

Figure S4. X-ray diffraction patterns at a Mg/SiO2 mole ratio of 4, 1273 K and various reaction times.

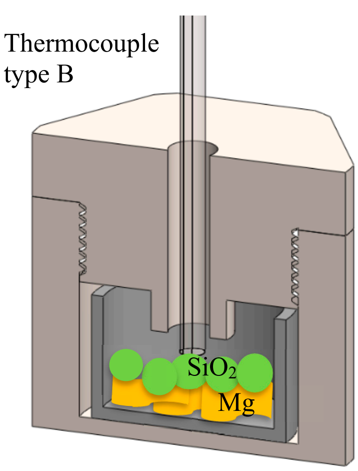

(a)

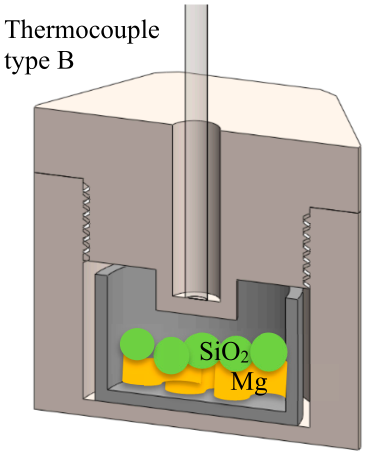

(b)

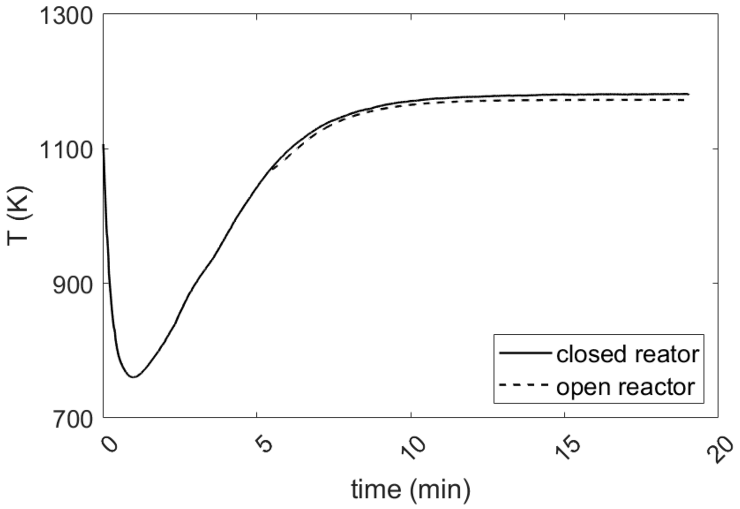

(c)

Figure S5. (**a**) Closed reactor used to conduct experiments; (**b**) open reactor to measure temperature close to reactant during a reduction reaction; (**c**) measured temperature against reaction time for two set-ups.

Table S2. Heat capacity of phases obtained from HSC Chemistry 9 software.

Phases	Temperature Range (K)	Heat Capacity (J/K.Kg)
SiO_2	298.15–847 (s)	$(58.082 - 0.033 \times 10^{-3}\times T - 14.259 \times 10^{5}\times T^{-2} + 28.221 \times 10^{-6}\times T^{2}/(0.060083)$
	847–1079 (s)	$(58.873 + 10.071 \times 10^{-3} \times T + 0.117 \times 10^{5} \times T^{-2})/(0.060083)$
	1079–1996 (s)	$(72.735 + 1.331 \times 10^{-3}\times T - 41.288 \times 10^{5} \times T^{-2} - 0.013 \times 10^{-6} \times T^{2})/(0.060083)$
	1996–4700 (l)	83.5/0.060083
MgO	298.15–1700 (s)	$(47.485 - 4.648 \times 10^{-3}\times T-10.34\times 10\ {}^{\wedge 5}\times T^{-2} - 0.268\times 10^{-6}\times T^{2})/(0.0403)$
	1700–3100 (s)	$(78.297 - 19.425 \times 10^{-3} \times T\text{-}171.03 \times 10^{5} \times T^{-2} + 5.156 \times 10^{-6} \times T^{2})/(0.0403)$
Mg_2Si	298.15–1373 (s)	$(73.304 + 14.979 \times T \times 10^{-3} - 8.828 \times T^{-2} \times 10^{5})/0.0766$
	1373-2000 (l)	(94.14)/0.0766

Table S3. Density and thermal conductivity of phases obtained from FactSage 8.1 software.

Temperature (K)	Phases properties			
	MgO		SiO_2	
	Density (kg/m^3)	Thermal Conductivity (W.m^{-1}K^{-1})	Density (kg/m^3)	Thermal Conductivity (W.m^{-1}K^{-1})
1050	3475.90	16.31	2554.10	4.48
1060	3474.30	16.16	2552.60	4.44
1070	3472.70	16.01	2551.20	4.39
1080	3471.20	15.87	2549.70	4.36
1090	3469.60	15.72	2548.30	4.32
1100	3468.00	15.58	2546.80	4.28
1110	3466.40	15.45	2545.40	4.24
1120	3464.80	15.31	2543.90	4.20
1130	3463.20	15.18	2542.40	4.17
1140	3461.70	15.05	2540.90	4.13
1150	3460.10	14.92	2235.90	1.08
1160	3458.50	14.79	2238.10	1.07
1170	3456.90	14.67	2240.30	1.06
1180	3455.30	14.55	2242.60	1.05
1190	3453.70	14.43	2245.00	1.04
1200	3452.10	14.31	2247.50	1.03
1210	3450.50	14.19	2250.00	1.02
1220	3448.90	14.08	2252.60	1.01
1230	3447.30	13.97	2255.20	1.00
1240	3445.60	13.86	2257.90	0.99
1250	3444.00	13.75	2260.70	0.99
1260	3442.40	13.64	2263.50	0.98
1270	3440.80	13.54	2266.40	0.97
1280	3439.20	13.43	2269.40	0.96
1290	3437.50	13.33	2272.40	0.95
1300	3435.90	13.23	2275.60	0.95
1310	3434.30	13.13	2278.70	0.94
1320	3432.70	13.03	2282.00	0.93
1330	3431.00	12.94	2285.30	0.92
1340	3429.40	12.84	2288.60	0.92
1350	3427.80	12.75	2292.10	0.91
1360	3426.10	12.66	2295.60	0.90
1370	3424.50	12.57	2299.20	0.89
1380	3422.80	12.48	2302.80	0.89
1390	3421.20	12.39	2306.50	0.88
1400	3419.50	12.31	2310.30	0.87
1410	3417.90	12.22	2314.20	0.87
1420	3416.20	12.14	2318.10	0.86
1430	3414.60	12.05	2322.10	0.85
1440	3412.90	11.97	2326.10	0.85
1450	3411.30	11.89	2330.30	0.84
1460	3409.60	11.81	2334.50	0.84
1470	3407.90	11.73	2338.80	0.83

1480	3406.30	11.66	2343.10	0.82
1490	3404.60	11.58	2347.50	0.82
1500	3402.90	11.50	2352.00	0.81
1510	3401.30	11.43	2356.60	0.80
1520	3399.60	11.36	2361.30	0.80
1530	3397.90	11.28	2366.00	0.79
1540	3396.30	11.21	2370.80	0.79
1550	3394.60	11.14	2375.70	0.78
1560	3392.90	11.07	2380.60	0.78
1570	3391.20	11.00	2385.60	0.77
1580	3389.50	10.94	2390.70	0.77
1590	3387.80	10.87	2395.90	0.76
1600	3386.10	10.80	2401.20	0.75
1610	3384.50	10.74	2406.50	0.75
1620	3382.80	10.67	2411.90	0.74
1630	3381.10	10.61	2417.40	0.74
1640	3379.40	10.55	2423.00	0.73
1650	3377.70	10.49	2428.60	0.73
1660	3376.00	10.42	2434.30	0.72
1670	3374.30	10.36	2440.10	0.72
1680	3372.60	10.30	2446.00	0.71
1690	3370.90	10.24	2452.00	0.71
1700	3369.10	10.19	2458.10	0.71
1710	3367.40	10.13	2464.20	0.70
1720	3365.70	10.07	2470.40	0.70
1730	3364.00	10.01	2476.70	0.69
1740	3362.30	9.96	2483.10	0.69
1750	3360.60	9.90	2489.60	0.68
1760	3358.80	9.85	1917.50	2.45
1770	3357.10	9.79	1913.90	2.44
1780	3355.40	9.74	1910.20	2.43
1790	3353.70	9.69	1906.50	2.42
1800	3351.90	9.64	1902.80	2.41
1810	3350.20	9.59	1899.10	2.39
1820	3348.50	9.53	1895.40	2.38
1830	3346.70	9.48	1891.60	2.37
1840	3345.00	9.43	1887.90	2.36
1850	3343.30	9.38	1884.20	2.35
1860	3341.50	9.34	1880.40	2.34
1870	3339.80	9.29	1876.70	2.33
1880	3338.00	9.24	1872.90	2.32
1890	3336.30	9.19	1869.10	2.30
1900	3334.50	9.15	1865.40	2.29
1910	3332.80	9.10	1861.60	2.28
1920	3331.00	9.05	1857.80	2.27
1930	3329.30	9.01	1854.00	2.26
1940	3327.50	8.96	1850.20	2.25
1950	3325.80	8.92	1846.40	2.24
1960	3324.00	8.88	1842.60	2.23

1970	3322.30	8.83	1838.70	2.22
1980	3320.50	8.79	1834.90	2.21
1990	3318.70	8.75	1831.10	2.20
2000	3317.00	8.70	2335.00	-
2010	3315.20	8.66	-	-
2020	3313.40	8.62	-	-
2030	3311.70	8.58	-	-
2040	3309.90	8.54	-	-
2050	3308.10	8.50	-	-
2060	3306.30	8.46	-	-
2070	3304.50	8.42	-	-
2080	3302.80	8.38	-	-
2090	3301.00	8.34	-	-
2100	3299.20	8.30	-	-
2110	3297.40	8.27	-	-
2120	3295.60	8.23	-	-
2130	3293.80	8.19	-	-
2140	3292.00	8.16	-	-
2150	3290.20	8.12	-	-
2160	3288.50	8.08	-	-
2170	3286.70	8.05	-	-
2180	3284.90	8.01	-	-
2190	3283.10	7.98	-	-
2200	3281.30	7.94	-	-
2210	3279.40	7.91	-	-
2220	3277.60	7.87	-	-
2230	3275.80	7.84	-	-
2240	3274.00	7.81	-	-
2250	3272.20	7.77	-	-
2260	3270.40	7.74	-	-
2270	3268.60	7.71	-	-
2280	3266.80	7.67	-	-
2290	3265.00	7.64	-	-
2300	3263.10	7.61	-	-
2310	3261.30	7.58	-	-
2320	3259.50	7.55	-	-
2330	3257.70	7.52	-	-
2340	3255.80	7.49	-	-
2350	3254.00	7.46	-	-
2360	3252.20	7.43	-	-
2370	3250.30	7.40	-	-
2380	3248.50	7.37	-	-
2390	3246.70	7.34	-	-
2400	3244.80	7.31	-	-
2410	3243.00	7.28	-	-
2420	3241.20	7.25	-	-
2430	3239.30	7.22	-	-
2440	3237.50	7.19	-	-
2450	3235.60	7.17	-	-

2460	3233.80	7.14	-	-
2470	3231.90	7.11	-	-
2480	3230.10	7.08	-	-
2490	3228.20	7.06	-	-
2500	3226.40	7.03	-	-
2510	3224.50	7.00	-	-
2520	3222.70	6.98	-	-
2530	3220.80	6.95	-	-
2540	3219.00	6.92	-	-
2550	3217.10	6.90	-	-
2560	3215.20	6.87	-	-
2570	3213.40	6.85	-	-
2580	3211.50	6.82	-	-
2590	3209.60	6.80	-	-
2600	3207.80	6.77	-	-
2610	3205.90	6.75	-	-
2620	3204.00	6.72	-	-
2630	3202.10	6.70	-	-
2640	3200.30	6.67	-	-
2650	3198.40	6.65	-	-
2660	3196.50	6.63	-	-
2670	3194.60	6.60	-	-
2680	3192.80	6.58	-	-
2690	3190.90	6.56	-	-
2700	3189.00	6.53	-	-
2710	3187.10	6.51	-	-
2720	3185.20	6.49	-	-
2730	3183.30	6.47	-	-
2740	3181.40	6.44	-	-
2750	3179.50	6.42	-	-
2760	3177.60	6.40	-	-
2770	3175.70	6.38	-	-
2780	3173.80	6.36	-	-
2790	3172.00	6.33	-	-
2800	3170.10	6.31	-	-
2810	3168.10	6.29	-	-
2820	3166.20	6.27	-	-
2830	3164.30	6.25	-	-
2840	3162.40	6.23	-	-
2850	3160.50	6.21	-	-
2860	3158.60	6.19	-	-
